# The Thailand Cave Rescue: General Anaesthesia in Unique Circumstances Presents Ethical Challenges for the Rescue Team

**DOI:** 10.1007/s11673-022-10168-w

**Published:** 2022-02-14

**Authors:** Mark A. Irwin

**Affiliations:** Bioethics Centre, University of Otago, P.O. Box 56, Dunedin, 9054 New Zealand

**Keywords:** Bioethics, Cave rescue, Disaster ethics, Anaesthesia

## Abstract

In 2018, the remarkable rescue of twelve young boys and their football coach trapped in a flooded cave in Thailand captured worldwide attention. The rescue required the boys to be dived out of the cave system while fully anaesthetized which presented unique practical and ethical challenges for the rescue team. Major departures from normal anaesthetic practice were required. Taking anaesthetized children underwater was unprecedented, complex, and dangerous. To do this underground in a flooded cave meant the risks were extreme. Using a principlist approach, this essay will outline the rescue plan highlighting the ethical dilemmas faced by the rescue team. Informed consent and full disclosure of information are justifiably waived in emergency disaster scenarios. Beneficence as a guiding principle becomes a major challenge when all rescue options appear destined to cause likely fatalities of healthy young boys. Importantly, virtues and virtue ethics also have a vital role to play when confronting and dealing with ethical challenges in disaster scenarios—this will be discussed with particular reference to the cave rescue.

## Introduction

In July 2018, twelve young boys aged eleven to sixteen and their twenty-five-year-old football coach were trapped deep underground in a flooded cave system in northern Thailand after being caught out by heavy rain. The story of their remarkable rescue received unprecedented global media coverage at the time.

The rescue team were a group of experienced international cave divers. Two of these divers were Australians with medical backgrounds: Dr. Richard Harris, an anaesthetist, and Dr. Craig Challen, a veterinarian. They had been approached to join the rescue team when it was realized that medical as well as diving expertise was required; extracting the boys from the cave would ultimately require them to be anaesthetized in extremely challenging circumstances. These two men, particularly Harris, took primary responsibility for deciding what form the rescue should take, the detailed management of the rescue plan, and the final decision to go ahead. It was a complex operation with extreme risks. This essay will outline the rescue plan highlighting the ethical challenges that were faced by the medical rescue team in these unique circumstances.

## Disasters, Ethical Approaches

In some respects, the cave rescue can be considered in the context of a disaster scenario. Disasters can take many forms, result from natural causes or be man-made, and can be defined in many ways. A good definition comes from the Centre for Research on the Epidemiology of Disasters (CRED) in Brussels, Belgium: “A disaster is a situation or event which overwhelms local capacity, necessitating a request to a national or international level for external assistance” (Zack 2009, 4).

Much of the literature discussing disaster management and associated ethical considerations centres on issues related to triage and allocation of scarce or inadequate resources. Utilitarian ethical models (saving the greatest number) predominate in discussions of real or hypothetical scenarios (lifeboat ethics).

The cave rescue, however, was a unique and unprecedented disaster scenario. Neither triage nor resource allocation were an issue. The primary challenge for the medical specialists and cave divers was to devise a plan to extract a group of young boys trapped underground in some of the most challenging circumstances imaginable.

The “Four Principles” approach to ethics in modern healthcare, first proposed by Beauchamp and Childress, has underpinned medical ethical practice in the western world for the last four decades; for example, the principles are referenced in both the Australian and New Zealand codes of medical ethics. They would have been the familiar ethical principles underlying the daily practice of the medical rescue team throughout their professional careers and would be the ethical framework they would take with them into the cave to help guide their decision making for the rescue.

The importance of sound foundational moral and ethical principles is highlighted in emergency situations where responders can find themselves under immense pressure. There is evidence that stress can specifically alter ethical decision-making, decrease consistency between belief and actions, and predispose to more risky decisions (Ryus and Baruch 2018). “Moral principles of normal times” should not be overridden in response to disaster (Zack 2009, 64). It is not a time to invent new codes of ethical conduct. It is interesting to note a comment made to Harris by his wife (also a physician) before his departure to Thailand to join the rescue team: “You’re the doctor there. These boys are your patients. It’s business as usual. It’s just that you’re in a very strange environment” (Challen and Harris 2019, 92). This very much captures the essence of this concept of keeping the same approach, practically and ethically, as much as possible.

It is therefore appropriate to consider the ethical challenges Harris and Challen faced using a principlist approach. Balancing considerations of beneficence, commonly reflected in the risks versus benefits judgements that are part of everyday medical practice, is a good basis to consider the challenges faced by the cave rescue divers. Conflicts regarding respect for autonomy will also be highlighted.

Finally, the important role and contribution virtue ethics also played when confronting and dealing with these challenges will be discussed.

The ultimate goal was clear—to get the boys out of the cave alive and to cause as little harm as possible while doing so.

## General Anaesthesia, Autonomy, and Beneficence

It had become clear very early in the rescue planning that to get the boys out of the flooded cave some form of diving rescue would be required. Teaching the boys to dive was not an option and it was clear to Harris and Challen that for safety reasons the boys would need to be fully unconscious to dive them out, with experienced cave divers taking them one at a time. The only practical way to achieve this was with general anaesthesia. It is reported in many accounts of the rescue that the boys were sedated; this was not the case and the distinction is important. Sedation implies some remaining and variable level of consciousness with attendant risks of disorientation, panic, flailing limbs, and mask dislodgement—all of which could quickly have had fatal consequences for the boys and possibly also the rescue divers. The rescue would require the boys to be fully unconscious with a general anaesthetic which is a significant medical intervention. As a specialist anaesthetist as well as an experienced cave diver, responsibility now shifted largely to Harris to plan and lead the rescue attempt. He had able support from his close friend and fellow diver Challen who had previous experience with veterinary anaesthesia and so a good understanding of the issues involved.

A comprehensive description of the technical details of the rescue, including details of the anaesthesia, has been published elsewhere (van Waart et al. 2020). For this discussion, it is important to consider the ways in which the unique circumstances of the rescue mandated significant variations to what would be normal practice in the provision of an anaesthetic to a healthy child.

These variations raised a number of issues: questions relating to autonomy including informed consent and disclosure and considerations of beneficence when significant life-threatening risks were unavoidable. These extreme risks would make subsequent moral conflicts very challenging.

Informed consent for a medical procedure, including anaesthesia, is standard practice in western medical practice and is the preeminent example of the principle of respecting the autonomy of a patient. In normal practice, certainly in Australasia, an anaesthetist would expect to discuss the conduct and risks of an anaesthetic with a young child’s parent or guardian before proceeding. Children the age of these boys would be involved in this discussion as appropriate for their age and level of understanding.

There are valid justifications for waiving the requirement for informed consent in certain circumstances. An emergency is the primary example where simple practicalities may render it impossible to follow a standard consent process. In these situations, paternalistic beneficence may have to take priority over rights of autonomy especially in life threatening circumstances. This is well recognized—for example both the New Zealand Medical Council and the Australian Medical Association have clauses in their respective codes of ethics recognizing the right to waive the requirement for consent in emergency situations.

This was certainly the case for the Thai cave rescue. Normal consent process was clearly not possible for many reasons including extreme time pressure, language and cultural barriers, the difficulties of dealing with a group rather than an individual and further complicated by the fact that these were children, separated and without guidance from parents and family. Involvement of the parents (or boys) in any attempted formal consent process could easily have jeopardized the entire rescue attempt, particularly if some agreed with a proposed plan and others did not. The situation has some parallels with public health ethics where policy is made considering the overall interests of a community rather than individuals.

The rescue team had no contact at all with family members of the boys and interestingly there were no approaches from family requesting information about rescue details. Family were told by the Thai authorities simply that a dive rescue was to be undertaken (Richard Harris, pers. comm.). It is interesting to speculate whether parents from other countries or cultures might have had different expectations about their inclusion in any discussions and the information that they would expect to receive, even if formal consent was not being sought.

When the rescue plan had been formulated, the boys were given only a very simple factual description, via an interpreter, of what was going to be required of them for the rescue. Risks were not mentioned and they were told only as much as it was felt they needed to know to enable the rescue to proceed. Conflicts involving considerations of beneficence and autonomy are not uncommon in normal medical practice and sometimes limited or non-disclosure of information is the appropriate course of action. This could certainly be justified in this unique situation. It is simply not possible to administer anaesthesia to children of this age group without their full cooperation, and this would be especially true in such extreme circumstances. Minimizing fear and anxiety was crucial and in his own account Harris describes watching the boys’ faces carefully for their reactions as the interpreter explained the rescue process. Gaining and maintaining trust between the boys and the rescue team was vital.

There were other more practical aspects that necessitated major departures from normal anaesthetic practice, the most obvious one being the requirement to take anaesthetized patients underwater. There was simply no precedent for doing this. During World War II in the United Kingdom studies had been performed on unconscious subjects in water while testing life jacket design but not involving sustained immersion (Challen and Harris 2019).

Some aspects of the anaesthetic technique warrant more detailed explanation. The boys would have tightly fitting, full face masks with a positive pressure oxygen supply but no other breathing support or airway devices. Maintaining a patent airway so that patients can breathe adequately, is one of the most fundamental tenets of anaesthesia. All general anaesthetics can to some degree impair breathing, cause muscle relaxation resulting in airway obstruction, impair protective reflexes with a risk of aspiration, and sometimes heighten other reflexes causing airway closure and hypoxia. All these complications can be harmful or even fatal if untreated. Preventing, recognizing, and dealing with these issues before they become a problem is part of normal daily practice for an anaesthetist. While anaesthetized these boys were to have unprotected, unmonitored, out of sight, and totally inaccessible airways for significant periods of time—and be underwater. This is breaking some very fundamental rules and would cause immense anxiety for any anaesthetist taking responsibility. Any ingress of water into the mask, certainly mask dislodgement, would be likely to have fatal results.

The proposed plan, a diving rescue involving anaesthetized children, was not remotely like anything that had ever been attempted before and the risk of complications with potentially fatal outcomes was extremely high. As best they were able, the rescue team thought through the practicalities and details of the plan including testing the masks and equipment on similar sized boys (not anaesthetized) in a local swimming pool the day before. This, however, was a world away from the reality of the conditions that would be faced. The trapped boys were two and a half kilometres underground inside a complex cave system, over one kilometre of which was fully flooded in sections which would require seven separate dives. The water was turbid with essentially zero visibility and there were multiple tight restrictions to get the boys through.

Compounding the risks was the requirement to hand over care of a fully anaesthetized child to non-medically trained assistants. After induction of anaesthesia by Harris, the boys were to be dived out one at a time by cave divers none of whom had any formal medical background apart from Challen. The prolonged time for each individual rescue (estimated three hours) meant that repeat intramuscular doses of anaesthetic would need to be given at intervals to keep the boys fully unconscious and avoid the problems listed earlier when discussing sedation. Anaesthesia training for the divers who would administer these top-up injections consisted of a brief talk from Harris and practice injecting into a plastic bottle, the day before the rescue was planned to begin.

Putting all this together, the risks of the proposed plan to dive the boys out were incalculably high. Also, it was not just one rescue—it would have to be repeated twelve times with the same level of risk each time. In his own mind, Harris wondered if they were crazy to even contemplate it (Challen and Harris 2019).

Were there other options? The only other possible option might be to leave the boys and their coach in the cave with adequate supplies until the monsoon season was over, water levels had dropped, and they could walk out as they had walked in. It would probably mean a minimum of four or five months in the cave. This option was seriously considered early on and was discussed at length. However, it would have presented insurmountable challenges. Issues would include getting enough supplies into the cave and room to store them, a fresh water supply, keeping warm and dry, sanitation, and inevitable medical problems such as dysentery not to mention mental health issues. The boys were perched on a damp, muddy slope in an air pocket which would probably get smaller with rising water. Hypoxia, literally running out of oxygen, may also have been an issue with a prolonged stay. A measurement of oxygen levels where the boys were trapped showed a reading of 15 per cent although this was not verified. Normal oxygen levels in air are 21 per cent.

When Harris first saw for himself the actual physical surroundings where the boys were trapped, he realized that this option was completely out of the question: “Leaving the boys there would have meant certain death, and an unpleasant and terrifying one at that” (Richard Harris, pers. comm.).

On the face of it, there seemed to be only one course of action to take and that was to continue with the plan to dive the boys out despite the very real risk of fatalities. Challen states this quite frankly in their account: “Both Harry (Harris) and I and everyone else involved in this plan fully expected that at least some of these children would die” (Challen and Harris 2019, 178). Ethically, this is becoming a very difficult situation to be in. Zack expresses the dilemma well: “The morality of disaster is a cause for anguish because choices are presented that involve loss, no matter what is done” (Zack 2009, 36).

Privately, Harris was even more pessimistic than Challen’s statement implies. He thought it most likely that *all* the boys would die while trying to get them out, the risks were so great (Richard Harris, pers. comm.). This must have been a profoundly difficult and distressing dilemma. Being the medically qualified anaesthetist in the team, the final decision to go ahead would rest with him as he was the one who would be administering the anaesthesia. In his own words: “It could be like I’m euthanizing these boys” (Challen and Harris 2019, 178).

The choices seemed stark and had gone a long way beyond the familiar balancing risks and benefits that are part of everyday medicine. Beneficence is a broad guiding principle only and some very thoughtful specification is required to guide decision-making in such extreme circumstances. It would be very difficult to reconcile the principle of beneficence with any course of action likely to cause fatalities but it seemed that this was exactly the situation they faced. For Harris, it appeared to be becoming more a choice between a good death (dying while anaesthetized, unaware) versus a bad death (lingering, unpleasant death in a cave).

In normal anaesthetic practice the risk of death for an otherwise healthy child is vanishingly small. A child dying in an operating theatre is very rare and is almost always in the setting of major trauma, congenital abnormality, or some known life-threatening illness. The boys in the cave were healthy, normal children, in fact described as “healthy, happy and smiley” by the divers who first found them. The prospect of anaesthetizing a completely well, healthy person, particularly a child, with the expectation that they will (probably) never wake up is a scenario so completely removed from normal anaesthetic practice it would be extremely difficult for any anaesthetist to come to terms with.

All anaesthetics involve a degree of trust. Ultimately, the patient has no option but to place their trust in the anaesthetist to take care of them. In a situation where full informed consent is not possible this becomes particularly significant. An anaesthetist who proceeds knowing that death is a more than likely outcome is potentially committing a huge betrayal of that trust if the patient is unaware they are likely to die. As well as dealing with a fatality, this breach of trust adds further to the significant distress from a moral and professional point of view.

Also confronting Harris and Challen was not just the likely probability of fatalities but the question of how they would deal with the consequences of that outcome. The two difficult issues they would be faced with were whether to carry on with further rescue attempts and secondly what if anything to tell the other boys. Deaths would be incredibly hard to deal with, especially if repeated. Harris believed that he personally would have been unable to continue with the rescue if the first few boys had all died (Harris, pers. comm.).

Harris and Challen also discussed between themselves the question of what to tell the boys still in the cave if fatalities occurred. If the plan was to continue it was felt to be in the boys’ best interests for them not to know about any deaths. The reasons and justification were essentially the same as described earlier—paternalistic beneficence again justifying non-disclosure. Causing any extra distress and anxiety would only be counter-productive if the rescue was to continue. Trust and cooperation would quickly be lost and further rescue attempts would have to be abandoned. Challen expressed this well in his own words: “Sometimes, somebody just needs to take charge. This time, we would be the ones to assume responsibility—to decide, uncomfortably, on their behalf” (Challen and Harris 2019, 182).

The rescue operation began fifteen days after the boys had first entered the cave. It took three days to get all the boys, their coach, and all the supporting diving team out of the cave. Quite remarkably they all survived.

## Contribution of Virtue Ethics

Complementary to, and as equally important as moral principles, are moral virtues. Virtues are commendable character traits, universally good for all people and manifested in habitual action. Moral virtues concern the person, the moral agent, while moral principles are more about actions and the two can never be completely separated. Any moral event involves an agent, an act, circumstances, and consequences. Different ethical theories will focus on these different elements (Pellegrino 1995).

The cave rescue can be viewed in this context, as a moral event. Considerations of beneficence, as well as the practicalities of what was possible from a diving and an anaesthetic perspective, guided the determination of the best course of action. However, it was the virtues, the traits of character of the rescuers (the moral agents) that enabled the difficult decisions to be made and the rescue to proceed.

Virtues have always been a vitally important and essential part of professional medical practice. This concept linking virtues with professional duties dates back to Aristotle: “… the state of character which makes a person good and makes that person do his or her work well …” (Aristotle, *Nicomachean Ethics*, cited in Pellegrino 1995). Many different lists of virtues considered essential for health professionals have been proposed but they are usually all very similar with considerable overlap. Commonly mentioned virtues include compassion, benevolence, thoughtfulness, trustworthiness, honesty, and integrity.

Simple adherence to principles is not adequate to deal with the complexity of moral and ethical problems encountered in normal medical practice, and this is especially true when faced with emergency or disaster situations. Virtues become even more critically important. “In morally ambiguous extreme cases, we do well to rely on the character or virtues of those in positions to make decisions” (Zack 2009, 64). Virtues, in conjunction with sound moral principles, help guide decision-making and subsequent courses of action in extreme circumstances. It has been argued that virtues are of prime importance in these situations. Virtue based ethics may be more adaptable to rapidly changing circumstances in disaster situations than principles or rules (Larkin and Arnold 2003).

Some virtues may assume special importance in times of crisis such as diligence, courage, and integrity. The careful planning, the decision to proceed despite seemingly overwhelming risks, and the general conduct of the rescuers are surely excellent examples of these virtues in action.

It is interesting to reflect on other aspects of the rescue where virtues played an important role.

This group of a dozen international cave divers were an example of a highly functioning team. They had come together at very short notice, literally from all over the world: the United Kingdom, Canada, Finland, Denmark, and Australia. Some of them were meeting each other for the first time. Together, in the space of a few days, they planned and executed a complex and dangerous rescue and did so under immense pressure.

The dive rescue was planned with meticulous attention to detail and this was later acknowledged as a significant factor in the eventual successful outcome (Coombes 2019). Attention to detail was critical—methods and techniques were being attempted for which there were no precedents. The equipment and techniques to be used were checked, tested, and rehearsed as much as was possible to minimize risks. To some extent this would have been second nature for the dive team. Cave diving is an inherently risky sport and careful equipment checks and formulating a detailed plan would be standard practice. Anaesthesia is also a medical specialty where equipment checks and formulating plans are very much normal professional practice.

Establishing trust with the boys was vital as it would have been impossible to go ahead without their full cooperation. Despite cultural and language barriers, and very limited time, Harris managed to establish a degree of trust that enabled the rescue to proceed. The extreme risks were not explained to the boys but they were certainly old enough to appreciate the very precarious situation they were in. They were prepared to put their full trust in a total stranger.

An important issue, largely in the background, was the safety of the rescue divers themselves. They were a team of willing volunteers, highly skilled and experienced. Cave diving is inherently risky in the best circumstances but risks to the divers would be significantly increased by taking responsibility for an unconscious child during a technically difficult dive. There was no hesitation by any of the team in accepting these personal risks. Emphasizing the danger was the fact that a former Thai Navy SEAL diver had tragically died in the cave a few days earlier.

Adding additional stress were two significant and constant external pressures which the rescue team had to deal with. The first was time pressure—it was the beginning of the monsoon season and the boys had been caught out by the first of what would certainly be many more heavy rainfalls. It was nine days before the boys were even found and the chances of more heavy rain at any time were very high. Rising water levels would have made the rescue even more difficult or impossible. This created a real sense of urgency throughout the entire rescue period.

The second external pressure was the extraordinary media coverage. There was a huge media presence outside the cave and any snippets of information were literally going to a global audience of millions. This could have been hugely distracting. It reflects well on the professional integrity of the rescue team that they were able to keep focused on the task at hand despite this intense and constant scrutiny.

## Conclusion

At face value, it would seem there were no dilemmas and no choices to be made. Despite the risks, the dive rescue as planned was the only option available. It would seem a straightforward decision to go ahead.

This superficial view, however, does not acknowledge the complexities of the underlying issues, the moral dilemmas, and the moral distress experienced by those involved to get to this point. To actively initiate a procedure that was very likely to kill healthy boys would be profoundly difficult and take extraordinary belief and resolve that you were doing the right thing. It demonstrates extraordinary strength of character. Knowing, on an intellectual level, that it was the boys’ only chance does not make it any easier when they are standing expectantly in front of you.

There was in fact another option that was never raised. The rescue team could have walked away: “This is an impossible situation, the risks to everyone including the rescuers are incalculably high, there is nothing we can do.” Who would have blamed them?

Supererogatory acts, in an ethical context, are examples of moral excellence, acts that go beyond normal moral obligations and expectations. They are “… optional, exceed what the common morality of obligation demands, intentionally undertaken to promote the welfare interests of others, morally good and praiseworthy in themselves ...” (Beauchamp and Childress 2019, 46). The actions and conduct of the rescue team are a fine example.

The Thai cave rescue is an inspirational story of one of the most extraordinary cave rescues of all time. The rescue team were truly remarkable men.

## References

[CR1] Beauchamp TL, Childress JF (2019). *Principles of biomedical ethics*.

[CR2] Challen C, Harris R (2019). *Against all odds*.

[CR3] Coombes, R. 2019. Success of Thai cave rescue depended on meticulous planning and rehearsal to minimise risks. *British Medical Journal* 365: doi.10.1136/bmj.l4131. 10.1136/bmj.l4131 31175118

[CR4] Larkin GL, Arnold J (2003). Ethical considerations in emergency planning, preparedness, and response to acts of terrorism. Prehospital and Disaster Medicine.

[CR5] Pellegrino ED (1995). Toward a virtue-based normative ethics for the health professions. Kennedy Institute of Ethics Journal.

[CR6] Ryus C, Baruch J (2018). The duty of mind: ethical capacity in a time of crisis. Disaster Medicine and Public Health Preparedness.

[CR7] van Waart H, Harris RJ, Gant N (2020). Deep anaesthesia: the Thailand cave rescue and its implications for management of the unconscious diver underwater. Diving and Hyperbaric Medicine.

[CR8] Zack N (2009). *Ethics for disaster*.

